# Small airway dysfunction: a shared, early, measurable, and potentially treatable trait across COPD, asthma, and fibrotic lung disease

**DOI:** 10.3389/fmed.2026.1834906

**Published:** 2026-05-13

**Authors:** Cong Xie, Kai Yang, Weifeng Tang, Huijie Zhang, Hualiang Jin, Congcong Li, Zhen Gao, Ying Wei, Maimaititusun Yalikun, Jingcheng Dong

**Affiliations:** 1 Institute of Integrative Medicine, Fudan University, Shanghai, China; 2 Department of Integrative Medicine, Huashan Hospital, Fudan University, Shanghai, China; 3 Yuquan Hospital, School of Clinical Medicine, Tsinghua University, Beijing, China; 4 Department of Pulmonary and Critical Care Medicine, Affiliated Hangzhou First People’s Hospital, Westlake University School of Medicine, Hangzhou, China; 5 College of Traditional Chinese Medicine, Xinjiang Medical University, Ürümqi, China; 6 Kashi BGI South Xinjiang Precision Medicine Laboratory, Kashi City People’s Hospital, Kashgar, China

**Keywords:** asthma, chronic obstructive pulmonary disease, fibrotic interstitial lung disease, oscillometry, small airway dysfunction, treatable trait, ventilation heterogeneity

## Abstract

Small airway dysfunction (SAD) has emerged as a key but historically under-recognized component of chronic respiratory diseases, offering a potential explanation for the frequent mismatch between symptom burden and spirometric findings. Increasing evidence suggests that the distal airway compartment represents an early and clinically meaningful site of physiological disturbance across chronic obstructive pulmonary disease (COPD), asthma, and fibrotic interstitial lung disease (ILD). Characterized by elevated peripheral resistance, ventilation heterogeneity, and a tendency toward airway closure, SAD links distal pathology to gas trapping, dynamic hyperinflation, and activity-limiting dyspnea. In asthma, strong physiological and longitudinal data support SAD as a prevalent and clinically relevant phenotype associated with poor control and exacerbation risk, with partial reversibility through targeted therapy. In COPD, structural injury and loss of terminal bronchioles appear early and contribute to symptoms primarily through modifiable mechanical consequences such as hyperinflation. In fibrotic ILD, emerging structural and physiological studies indicate early distal involvement and distinct mechanical signatures, although evidence for therapeutic modification remains limited. Considered across diseases, SAD satisfies several key features of a treatable trait in that it is measurable, clinically meaningful, and closely connected to mechanisms that shape symptoms and functional limitation, even if its reversibility differs between conditions. Framing SAD within a “treatable trait” perspective may therefore provide a unifying approach to linking symptoms, physiology, and underlying pathology, and support more individualized strategies for assessment and management.

## 1 Introduction

Chronic respiratory diseases, including chronic obstructive pulmonary disease (COPD), asthma, and fibrotic interstitial lung disease (ILD), collectively represent a significant global health burden, contributing to substantial morbidity, healthcare resource utilization, and premature mortality (1). Despite notable advances in pharmacotherapy and biologics in recent years, a persistent clinical dilemma remains prominent: many patients experience significant dyspnea, reduced exercise tolerance, and increased risk of exacerbations even when spirometry reveals only mild abnormalities or appears largely normal by conventional measures (2, 3).

This widespread phenomenon of symptom–spirometry discordance underscores fundamental limitations of current diagnostic paradigms. Spirometry, centered on forced expiratory volume in 1 s (FEV_1_) and the FEV_1_/FVC ratio, has long served as the cornerstone for defining obstructive lung diseases, staging their severity, and guiding management according to clinical guidelines (4). However, spirometry is inherently insensitive to pathological processes occurring in the lung periphery. The small airways, bronchioles less than 2 mm in diameter and devoid of cartilaginous support, normally contribute only a minor fraction of total airway resistance. Consequently, even substantial peripheral airway pathology may remain undetected by conventional pulmonary function tests for prolonged periods. As a result, peripheral airway dysfunction has long been regarded as a “quiet zone,” systematically overlooked in both clinical practice and trial design (5).

Over the past decade, this traditional understanding has undergone a paradigm shift. In COPD, pathological and micro-CT studies have demonstrated that narrowing and loss of terminal bronchioles occur early in the disease course, even preceding the development of emphysema and the decline in spirometric indices (6). In asthma, large cohort studies such as the ATLANTIS study have further established small airway dysfunction (SAD) as a highly prevalent phenotype, strongly associated with poor disease control and susceptibility to exacerbations, even among patients with entirely normal spirometry (7, 8). More paradigm-challenging is the growing evidence indicating that fibrotic ILD, including idiopathic pulmonary fibrosis (IPF), is not solely an interstitial disorder: significant reduction in terminal bronchioles has been observed even in regions with minimal fibrosis (9, 10), while oscillometry can detect peripheral mechanical abnormalities before overt airflow obstruction becomes apparent (11).

Together, these observations support a unifying concept: SAD represents not merely a secondary consequence of advanced disease, but rather a shared, early, and clinically meaningful pathophysiological trait across chronic lung disorders. Importantly, SAD is increasingly measurable through sensitive physiological and imaging tools, and may constitute a modifiable target through therapies optimized for distal airway deposition and mechanistic intervention.

Thus, the central question addressed in this review is whether SAD constitutes an early, measurable, and treatable trait shared by COPD, asthma, and fibrotic ILD, thereby explaining the discordance between symptoms and spirometry while also predicting key clinical outcomes such as exacerbations, functional decline, and disease progression (12). As a key conceptual contribution, this review introduces an integrated “terminal ventilatory unit” framework that unifies SAD pathophysiology across COPD, asthma, and fibrotic ILD—encompassing terminal bronchioles, acinar structures, and their surrounding parenchymal matrix as a single functional entity.

## 2 Concepts and definitions

### 2.1 Functional SAD versus structural small airway disease

Small airways, typically defined as bronchioles with an internal diameter less than 2 mm and lacking cartilaginous support, constitute the transitional zone connecting the conducting airways to the gas-exchanging acinar units (6). Although these airways comprise the vast majority of the airway branching network, their physiological contribution to total airway resistance is relatively minor under healthy conditions. This unique characteristic explains why significant pathology can accumulate in the peripheral airways over an extended period while conventional spirometric indices may remain normal, thereby reinforcing the traditional view of the lung periphery as a “silent zone” (13).

**Figure 1:**
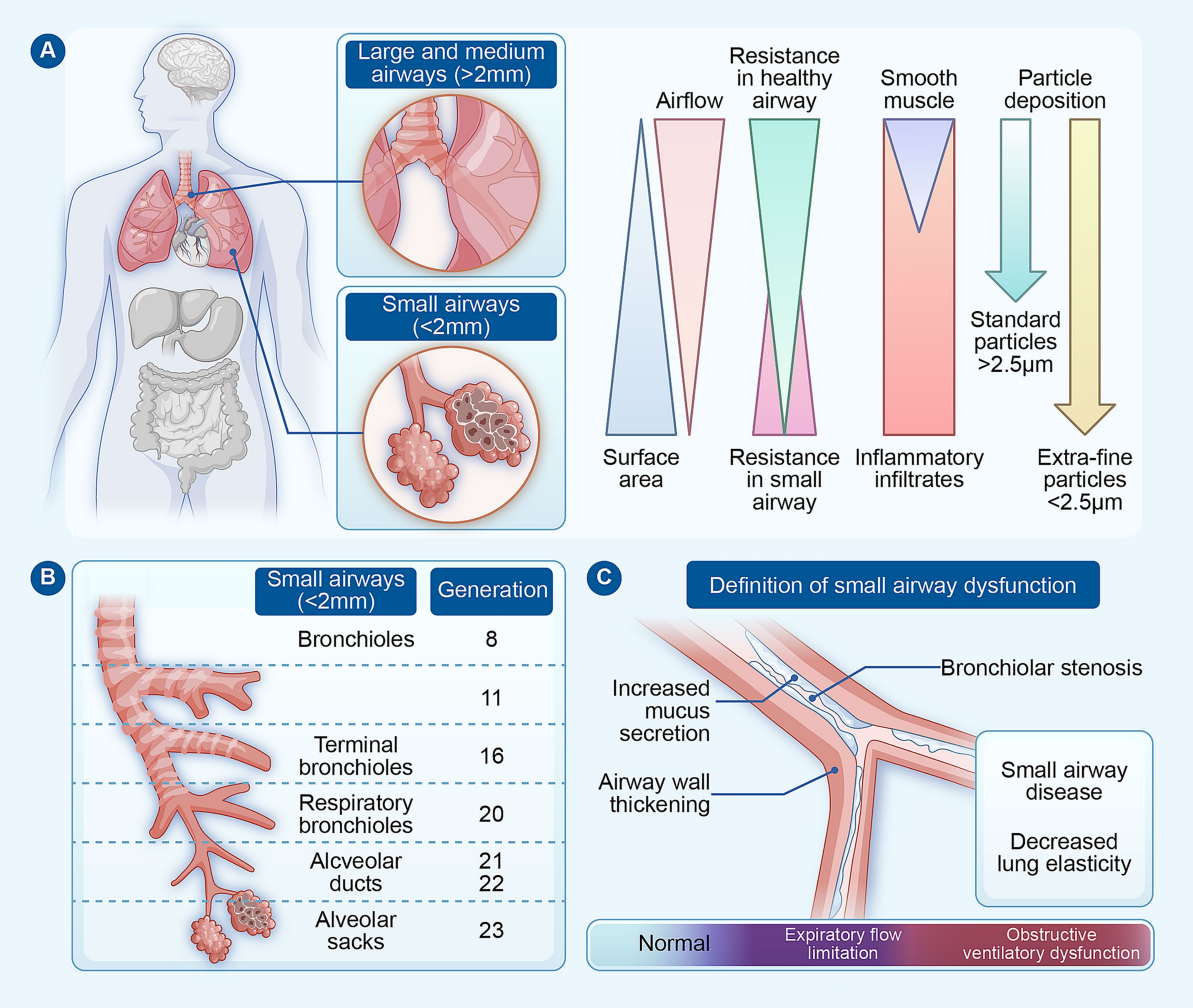
Definition and conceptual framework of small airways and small airway disease. **(A)** Comparison between large/central airways and small (peripheral) airways. Small airways are generally defined as bronchioles with an internal diameter < 2 mm and lacking cartilaginous support. Although they constitute the vast majority of the airway branching network and contribute minimally to total airway resistance under normal physiological conditions, they are characterized by lower airflow velocity, a large cumulative surface area, absence of cartilage, relatively sparse smooth muscle, and a microenvironment more prone to inflammatory infiltration and heterogeneous particle deposition. **(B)** Anatomical hierarchy of the small airway compartment. Small airways include membranous bronchioles, terminal bronchioles, and transitional bronchioles, which progressively lead into the acinar region. **(C)** Pathological and functional characteristics of SAD, showing how structural lesions lead to physiological abnormalities and clinical consequences despite normal conventional spirometry.

Within this context, the term “SAD” has gained traction to describe a functional phenotype characterized by mechanical abnormalities and ventilation distribution heterogeneity in the peripheral airways (14). SAD itself is not a pathological diagnosis but rather reflects a spectrum of measurable physiological disturbances detectable by specialized pulmonary function tests. These include: (1) frequency-dependent airflow limitation, where respiratory resistance increases as oscillation frequency decreases (measured by the difference between resistance at 5 Hz and 20 Hz, R5-R20); (2) reduced respiratory reactance at low frequencies (X_5_) and increased area of reactance (AX), reflecting peripheral airway closure and gas trapping; and (3) increased ventilation heterogeneity and enhanced propensity for airway closure during expiration (15) (Figure 1). Importantly, these functional abnormalities can emerge even before spirometry reveals overt obstruction, providing a physiological explanation for the clinically common observation that patients may experience significant symptom burden and elevated exacerbation risk despite FEV_1_ remaining within the normal range.

In contrast, “small airway disease” refers to the underlying structural pathological basis within the bronchiolar region. The spectrum of lesions includes inflammatory thickening of the airway wall, luminal narrowing, mucus plugging, peribronchiolar fibrosis, and even obliteration or loss of terminal bronchioles. In COPD, micro-CT studies have definitively established that the loss of terminal bronchioles is an early and central pathological event, occurring even before the development of emphysema and the decline in spirometric measures (6, 16). This finding challenges the traditional notion that peripheral airway injury is merely a secondary consequence of parenchymal destruction. In asthma, distal airway remodeling similarly contributes to persistent disease instability and is strongly associated with poor control in patients with normal lung function (17). More recently, evidence from fibrotic ILD has further disrupted classical compartmental thinking, as *in vivo* optical coherence tomography has revealed marked bronchiolar abnormalities and reductions in bronchiole number even in minimally fibrotic lung regions (10, 18).

A key concept is that functional SAD and structural small airway disease are complementary yet non-interchangeable domains. Similar physiological features may correspond to vastly different pathological mechanisms across diseases, and significant bronchiolar damage may remain functionally silent until a critical threshold is reached. Recognizing this incomplete functional-structural correspondence is fundamental for understanding why identical physiological parameters may manifest differently in COPD, asthma, and fibrotic ILD, and ultimately for positioning SAD as a *trans*-disease trait rather than a single disease marker.

### 2.2 The “terminal ventilatory unit” framework

**Figure 2:**
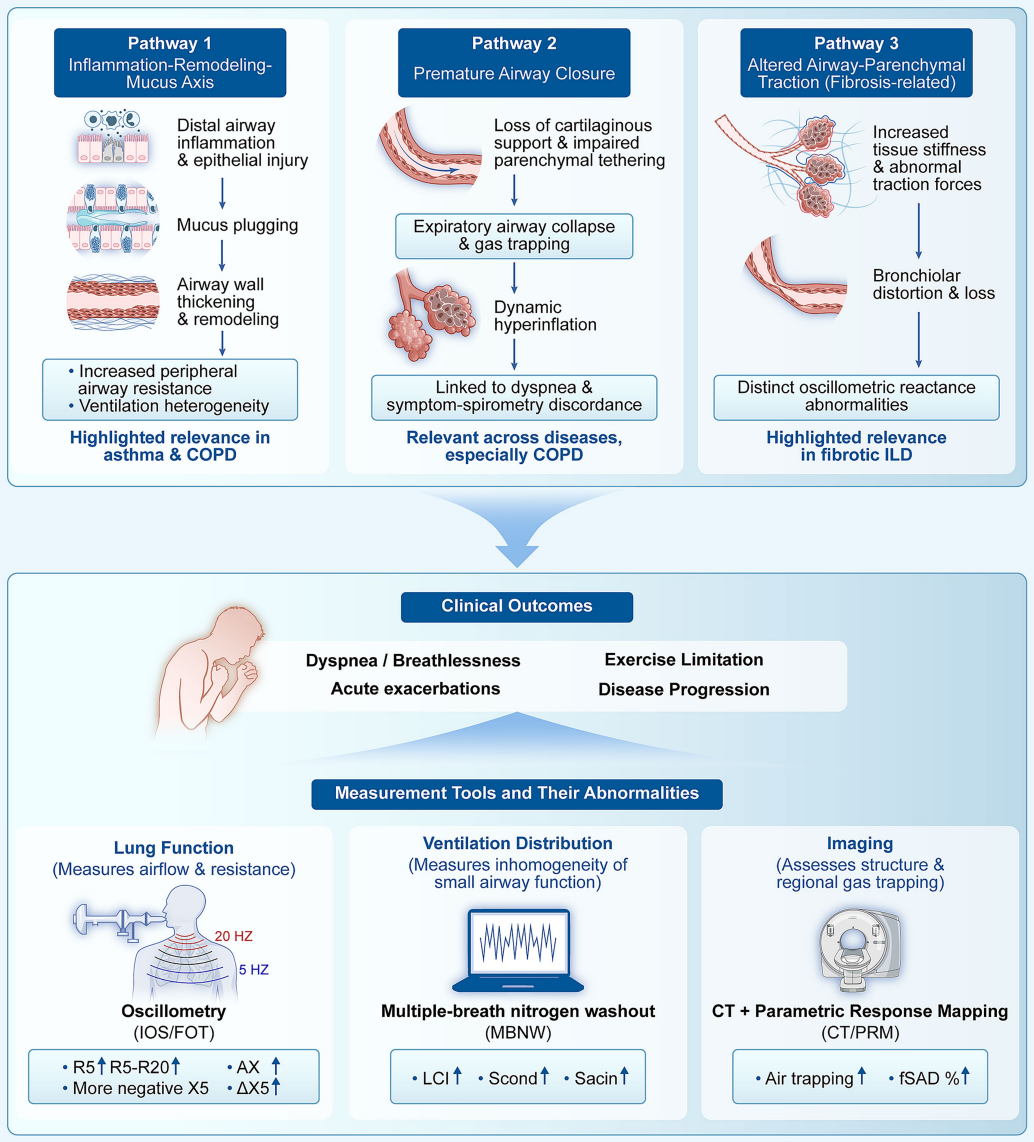
Mechanisms, clinical manifestations, and assessment of small airway dysfunction. The inflammation–remodeling–mucus axis, premature airway closure, and altered airway–parenchyma mechanical coupling collectively drive distal airway abnormalities, manifesting as clinical outcomes such as dyspnea, exercise limitation, exacerbations, and disease progression. Oscillometry, multiple-breath nitrogen washout, and imaging-based approaches capture these measurable physiological phenotypes from complementary perspectives.

Despite growing recognition of its clinical importance, there remains no universally accepted physiological definition or standardized diagnostic threshold for SAD, which limits cross-study comparability and clinical implementation. To address this gap, we propose an integrated “terminal ventilatory unit” framework that unifies SAD pathophysiology across diseases, encompassing terminal bronchioles, acinar structures, and their surrounding parenchymal matrix as a single functional entity. Within this unit, three mechanistic pathways appear to dominate the development and clinical expression of SAD (Figure 2).

Within the “terminal ventilatory unit” framework, three key pathophysiological mechanisms driving SAD are presented. Pathway 1, the inflammation-remodeling-mucus axis, primarily leads to increased peripheral airway resistance and ventilation heterogeneity through structural airway changes and obstruction. Pathway 2, premature airway closure, highlights mechanical instability, where the loss of structural support causes expiratory collapse and dynamic hyperinflation, establishing a direct physiological link between distal dysfunction and dyspnea. Pathway 3, altered airway-parenchymal traction, reflects increased tissue stiffness and abnormal traction due to fibrosis, resulting in bronchiolar distortion and unique mechanical abnormalities. These underlying pathophysiological alterations ultimately translate into physiological features that can be quantified by specific assessment tools, collectively driving adverse clinical outcomes.

The first shared axis is inflammation-driven remodeling and mucus-related obstruction, which together increase distal airway resistance and generate ventilation heterogeneity. In asthma, small airway inflammation, epithelial injury, mucus production, and airway wall remodeling are repeatedly identified as core mechanisms leading to increased peripheral resistance and airway instability (14). These distal abnormalities propagate downstream as ventilation heterogeneity and contribute to airway hyperresponsiveness, even while spirometric indices remain preserved (19). In COPD, the corresponding distal regions are similarly characterized by chronic inflammation and structural remodeling, but additionally bear the burden of bronchiolar narrowing and progressive loss of terminal bronchioles. These changes increase peripheral resistance while promoting uneven lung emptying, gas trapping, and the formation of regional air trapping, long before emphysema becomes a dominant imaging feature (6). Importantly, emerging models of IPF increasingly incorporate airway epithelial injury and distal airway structural abnormalities into an “airway-centric” framework, supporting a new paradigm in which airway pathology is not merely a secondary manifestation of interstitial fibrosis but may participate in the early evolution of the disease (9).

A second mechanistic pathway is premature airway closure, which provides a direct physiological bridge between distal dysfunction and the cardinal patient experience of exertional dyspnea (14). Because small airways lack cartilaginous support and are highly dependent on parenchymal tethering forces, they are uniquely vulnerable to collapse when structural integrity is compromised. In COPD, dynamic hyperinflation during exercise is highly prevalent and has profound mechanical and sensory consequences, including neuromechanical dissociation, constrained tidal volume expansion, and activity-limiting breathlessness (20). Premature distal airway closure during expiration represents a plausible contributor to this phenomenon, linking functional SAD to symptom burden in a manner that is often poorly captured by FEV_1_ severity staging. In asthma, airway closure and regional ventilation defects, frequently associated with mucus plugging and distal obstruction, are increasingly recognized as pathophysiologically central rather than epiphenomenal (21). Imaging studies have reinforced spatial relationships between ventilation defects, small airway closure, and air trapping, further positioning SAD as a mechanistic substrate for exacerbation-prone and poorly controlled phenotypes despite “normal” spirometry.

The third, and disease-defining, dimension occurs in fibrotic ILD, where altered parenchymal-airway traction coupling produces distinct mechanical fingerprints that may reflect more than obstructive narrowing (9). Fibrosis fundamentally changes tissue stiffness, airway tethering, and the balance of forces governing airway caliber and closure behavior (22). Consequently, peripheral airway mechanics in fibrosis may reflect a complex interplay between bronchiolar distortion, obliteration, and retraction by stiffened parenchyma (23), generating forced oscillation technique and impedance signatures distinct from classical obstructive disease patterns (11). Airway-centric models of IPF increasingly summarize distal airway structural abnormalities and propose an airway-driven contribution to fibrotic pathogenesis (9). In this context, endobronchial optical coherence tomography (EB-OCT) provides a direct *in vivo* structural anchor, demonstrating early bronchiolar dropout and distortion even in minimally fibrotic lung regions, thereby suggesting that terminal bronchiole loss can precede overt architectural remodeling (10). These observations motivate the hypothesis that oscillometry and other mechanics-based tools may capture a fibrosis-associated mechanical phenotype that is complementary to conventional longitudinal trends in FVC or DL_CO_.

## 3 Measurement toolbox

Having established SAD as a key pathological feature across diseases, a more practical question arises: can this trait be measured in a stable, reproducible, and clinically interpretable manner. If SAD is indeed a common early link connecting COPD, asthma, and fibrotic ILD, then its value lies not only in mechanistic explanation but also in whether it can serve as a quantifiable phenotype for identifying high-risk populations, explaining symptom-spirometry mismatch, and reflecting disease progression or treatment response during longitudinal follow-up (17, 24).

The limitations of traditional spirometry in this regard are already well-established. Since small airways contribute minimally to total airway resistance under normal physiological conditions, changes in FEV_1_ or FEV_1_/FVC only manifest when distal airway pathology becomes extensive and reaches a certain threshold (17). Consequently, if spirometry is used as the sole assessment tool, SAD is often only indirectly reflected at relatively advanced disease stages, failing to capture mechanical abnormalities in its early phases (25). This realization has driven the development of multiple novel assessment techniques over the past two decades (Table 1), which attempt to capture functional changes in the “terminal ventilatory unit” from different perspectives and establish connections with structural damage and clinical outcomes.

**Table 1:** Assessment techniques for SAD across physiological domains.

Method	Primary dimension	Typical SAD readout (examples)	Practical strengths	Key limitations
Oscillometry (IOS/FOT)	Respiratory impedance (resistance + reactance) during tidal breathing	R5-R20, X_5_, AX, Fres; ΔX5; within-breath indices	Effort-light; scalable; captures distal heterogeneity/closure signals; increasing accessibility in outpatient settings	Device/protocol heterogeneity; reference equations still evolving
Spirometry-derived indices	Airflow during forced expiration	FEF_25–75_, FEV_3_/FEV_6_ (with caveats)	Widely available; low marginal cost	High variability; effort and FVC-dependent; weak specificity for distal pathology
MBNW	Ventilation heterogeneity and gas mixing	LCI; S_cond_/S_acin_ (conductive vs. acinar heterogeneity)	Sensitive to early distal abnormality; mechanistically informative	Longer test time; analysis complexity; standardization hurdles
Inspiratory/expiratory CT; PRM	Structural + functional consequences (air trapping)	Air trapping metrics; PRM-fSAD vs. emphysema	Regional mapping; structure–function bridging	Radiation; protocol dependence; computational requirements
EB-OCT	*In-vivo* microscopic structure	Bronchiole density; stereology (shape, irregularity)	Direct structural anchor; early IPF signal	Invasive; limited availability; mainly research/tertiary centers

IOS, impulse oscillometry system; FOT, forced oscillation technique; X_5_, reactance at 5 Hz; AX, area of reactance; Fres, resonant frequency; ΔX5, inspiratory-expiratory difference in X_5_; MBNW, multiple-breath nitrogen washout; LCI, lung clearance index; PRM, parametric response mapping; EB-OCT, endobronchial optical coherence tomography.

### 3.1 Spirometry-derived indices

Given the ubiquity of spirometry, several indices derived from forced expiratory maneuvers have been proposed as surrogate markers of small airway function (4). The most widely used is the forced expiratory flow between 25 and 75% of forced vital capacity (FEF_25–75_; historically termed maximal mid-expiratory flow, MMEF), which has long been associated with distal airway pathology (26–29). However, contemporary ATS/ERS technical standards consistently urge caution in its interpretation, as FEF_25–75_ is strongly influenced by FVC measurement validity, expiratory effort, and lung volume, making it inherently variable and reducing its specificity for isolated distal disease. These limitations have led to recommendations that FEF_25–75_ should not be used as a stand-alone anchor for clinical decision making.

More recently, the ratio of forced expiratory volume in 3 s to forced expiratory volume in 6 s (FEV_3_/FEV_6_) has emerged as a more robust spirometric marker of early airway obstruction and SAD (30). Unlike FEF_25–75_, FEV_3_/FEV_6_ is less affected by incomplete exhalation and demonstrates better reproducibility across populations (31). In COPD, a reduced FEV_3_/FEV_6_ has been shown to predict future FEV_1_ decline and exacerbation risk in smokers with preserved FEV_1_/FVC ratios, identifying individuals with pre-obstructive disease characterized by isolated small airway dysfunction (32, 33). In asthma, abnormal FEV_3_/FEV_6_ correlates with oscillometric evidence of peripheral airway impairment and poor disease control, even in patients with normal FEV_1_.

Collectively, spirometry-derived indices should be interpreted as low-specificity screening signals that may complement a structured SAD phenotype rather than serve as definitive diagnostic tools (25). While they capture aspects of distal pathology in large cohort studies, their limited sensitivity and specificity mean that abnormal results should prompt further evaluation with more specialized techniques such as oscillometry.

### 3.2 Oscillometry

The recent ERS technical standards define oscillometry as the measurement of respiratory system impedance during quiet tidal breathing through externally applied pressure oscillations, producing resistance (Rrs, R) and reactance (Xrs, X) components that reflect airway and tissue mechanics (34). This conceptual shift from forced expiratory flows to impedance measured during tidal breathing is particularly relevant for distal airway phenotyping, as oscillometry is sensitive to frequency-dependent resistance changes and peripheral compliance alterations that are poorly captured by spirometry (35).

A key implication emphasized in the standards is that oscillometry is fundamentally different from traditional lung function tests. Variations in device hardware, signal processing algorithms, and breathing protocols can influence absolute values, making transparency and methodological standardization essential if SAD is to be interpreted as a trait across diseases and studies (34). The standards also highlight technical considerations directly relevant to distal airway assessment, including the need to perform oscillometry before deep-breath maneuvers such as spirometry or FeNO, given that lung volume history can acutely modify airway tone and closure behavior (36). Evidence-based bronchodilator response thresholds are summarized, with consensus-derived cut-offs of −40% relative change for R5, +50% relative improvement for X_5_, and −80% relative change for AX proposed to define physiologically meaningful responses, while acknowledging that device-specific validation remains incomplete.

Importantly, the oscillometric signature of SAD differs between asthma and COPD, reflecting distinct underlying pathophysiological mechanisms (37). In asthma, SAD is primarily characterized by increased frequency-dependent resistance (R5-R20) due to distal airway inflammation and smooth muscle constriction, whereas in COPD, abnormalities in reactance parameters (X_5_ and AX) are more prominent, reflecting premature airway closure, gas trapping, and parenchymal destruction (38). A recent international Delphi consensus in adults with asthma or COPD has established standardized z-score thresholds for identifying abnormal oscillometry: R5 > 1.64, X_5_ < −1.64, and AX > 1.64, with these thresholds indicating the presence of small airway dysfunction (39).

Of particular clinical relevance in COPD is the change in X_5_ from inspiration to expiration (ΔX5), which quantifies tidal expiratory flow limitation and dynamic hyperinflation. An established threshold of ΔX5 ≥ 0.28 kPa/L/s has been shown to strongly correlate with exercise intolerance, symptom burden, and exacerbation risk in patients with mild-to-moderate COPD (40–42). This index provides a direct physiological link between distal airway closure and the cardinal symptom of exertional dyspnea, offering prognostic information beyond conventional spirometric measures.

Among all currently available techniques, oscillometry has some of the strongest longitudinal outcome data in asthma (8). In the ATLANTIS cohort, a composite ordinal score integrating percent-predicted R5–R20, AX, and X_5_ independently predicted asthma control and future exacerbations even after adjustment for conventional predictors including GINA treatment step, prior exacerbations, blood eosinophil count, and FEV_1_ (8). Notably, when this composite physiological signal was included in multivariable models, the predictive contribution of FEV_1_ was attenuated, suggesting that oscillometry captures outcome-relevant distal physiology not adequately represented by spirometry (43, 44).

In COPD and tobacco-exposed populations, oscillometry-defined SAD has been increasingly linked to symptom burden and clinical outcomes (45), and treatable-trait–oriented cohort studies now explicitly evaluate IOS-defined abnormalities as potentially under-recognized drivers of morbidity in mild-to-moderate disease (38, 46). In fibrotic ILD, oscillometric reactance abnormalities have been associated with survival and prognostic indices, raising the possibility that impedance-derived parameters reflect disease-relevant distal heterogeneity even in the absence of classical obstruction (11, 47). Taken together, oscillometry appears to offer a scalable, effort-light method that captures closure-related mechanics and ventilation heterogeneity across diseases, making it a plausible front-line physiological marker of SAD (48, 49).

### 3.3 Multiple-breath nitrogen washout (MBNW)

Multiple-breath washout testing interrogates a different physiological dimension by quantifying ventilation heterogeneity and gas mixing inefficiency (50). Rather than measuring resistance, it captures how uniformly air distributes across lung regions during tidal breathing. The lung clearance index provides a global measure of gas mixing abnormality, while derived indices such as S_cond_ and S_acin_ attempt to separate conductive airway and acinar contributions to ventilation inhomogeneity (51).

Conceptually, this approach aligns closely with the terminal ventilatory unit framework. Distal airway closure, mucus obstruction, and uneven airway caliber all produce compartmental differences in filling and emptying, which manifest as abnormal washout kinetics. In asthma, abnormalities in acinar-level heterogeneity have been linked to disease instability and exacerbation propensity, particularly when combined with oscillometric evidence of peripheral mechanical dysfunction (52). In COPD, similar indices correlate with gas trapping and structural distal airway damage, supporting their role as mechanistic markers rather than purely descriptive physiology (53).

The value proposition of multiple-breath washout in a cross-disease SAD framework is therefore not to replace oscillometry, but to add mechanistic specificity. Where oscillometry provides a global mechanical signal, washout can help dissect whether heterogeneity arises primarily from conductive versus acinar compartments. In practice, this suggests a tiered approach in which oscillometry serves as a scalable screening tool, while washout is reserved for mechanistic phenotyping, early disease detection studies, and clinical trials seeking sensitive intermediate endpoints. Barriers remain, including longer test duration, repeatability criteria, and analytic complexity, which currently limit routine clinical implementation (50, 54).

### 3.4 Inspiratory/expiratory CT and PRM

Imaging provides a spatially resolved perspective on the consequences of distal airway dysfunction (55). Expiratory CT, in particular, allows air trapping and mosaic attenuation to be visualized as indirect markers of small airway obstruction and closure (56). The accuracy of these measures depends on acquisition quality, expiratory effort, and analytic methodology, but advances in automated computational analysis have improved detection and quantification (57).

A major step forward has been the development of parametric response mapping (PRM), which co-registers inspiratory and expiratory CT to classify lung tissue voxel by voxel into normal parenchyma, emphysema, and functional small airway disease (55). In COPDGene-based studies, PRM-defined fSAD (functional SAD) has been shown to precede emphysema development as disease severity increases, supporting a model in which distal airway dysfunction represents an early pathological stage (55). Longitudinal analyses further suggest that regional fSAD may evolve into emphysematous destruction over time, strengthening the argument that air trapping phenotypes reflect disease evolution rather than static severity (58).

In asthma, quantitative imaging markers including air trapping and airway remodeling have been associated with exacerbation risk and lung function trajectories (56), although their predictive role appears less consistent than that of scalable physiological measures. Indeed, some large cohorts have reported limited correlations between CT parameters and symptom control, underscoring a practical reality: imaging may be most valuable for regional phenotyping and mechanistic insight rather than routine risk prediction when physiological tools already perform strongly.

### 3.5 EB-OCT

The EB-OCT introduces a new level of structural resolution by enabling near-microscopic *in vivo* visualization of small airway architecture (59). Initially explored as a minimally invasive method to assess usual interstitial pneumonia patterns, EB-OCT has demonstrated high diagnostic accuracy relative to histopathology and clinical diagnosis in selected contexts, positioning it as a potential complement to HRCT and, in some cases, an alternative to surgical lung biopsy (59).

Most relevant to the concept of SAD as an early shared trait, recent studies have quantified small airway involvement in early IPF using EB-OCT (10). Defining small airways as structures with maximal luminal diameter ≤2 mm and sampling multiple bilateral regions, investigators observed substantial reductions in bronchiole density compared with matched controls (10). Importantly, bronchiolar loss was present not only in visibly affected regions but also in less affected areas, suggesting that distal airway dropout may precede overt architectural remodeling (10). Distortion of airway shape was more pronounced in fibrotic regions, while loss of bronchioles appeared relatively widespread, reinforcing the hypothesis that structural small airway involvement is an early component of fibrotic disease rather than a late complication (10).

These findings provide a critical structural anchor linking distal mechanical abnormalities to underlying pathology. They also strengthen the conceptual bridge between oscillometry-derived “mechanical fingerprints” and tissue-level changes, particularly in fibrotic ILD, where traditional functional measures such as FVC and DL_CO_ provide only indirect insight into distal heterogeneity.

## 4 Disease-specific evidence

**Figure 3:**
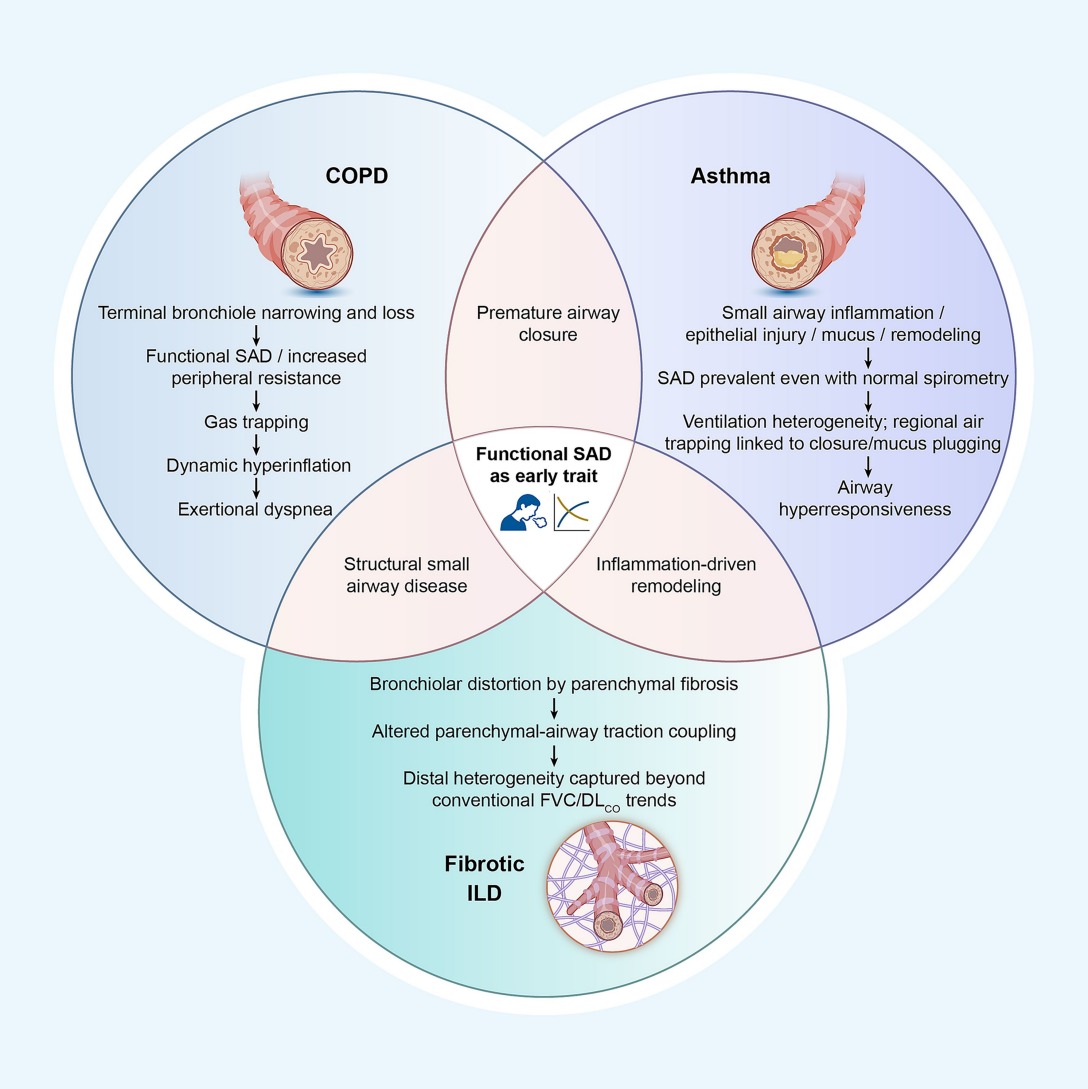
Small airway dysfunction (SAD) as a shared early functional trait across COPD, asthma, and fibrotic ILD. Functional SAD is characterized by premature airway closure, ventilation heterogeneity, and gas trapping. In COPD, structural changes in the small airways drive symptoms and disease progression. In asthma, distal inflammation and remodeling contribute to SAD, even in patients with normal spirometry. In fibrotic ILD, changes in airway-parenchymal traction lead to mechanical abnormalities in the small airways, affecting disease progression and symptom burden.

Once SAD is positioned as a cross-disease trait, the central question becomes whether this physiological phenotype carries consistent clinical meaning across conditions, and where it sits along the natural history of disease: as an early driver, a parallel mechanism, or a late consequence. If SAD truly represents a shared pathological link across COPD, asthma, and fibrotic ILD, then re-examining disease-specific evidence through a common analytical framework helps explain the long-recognized mismatch between symptoms and spirometry and supports its conceptualization as a candidate treatable trait (Figure 3).

### 4.1 COPD

Among chronic lung diseases, COPD provides the most compelling evidence that small airway pathology is not merely a secondary feature but an early and central lesion (16). Structural work using micro-CT has demonstrated that narrowing and loss of terminal bronchioles can precede the development of emphysema, establishing a plausible causal chain from distal airway injury to increased peripheral resistance and downstream parenchymal destruction (16). This concept was reinforced by studies showing marked loss of terminal and transitional bronchioles even in GOLD stage 1–2 disease, accompanied by thickened walls and luminal narrowing, indicating that bronchiolar pathology may occur at a stage when alveolar surface area can remain relatively preserved (60).

These structural findings align with a long-standing clinical observation: symptoms often precede diagnostic spirometric thresholds by many years (61). Current diagnostic frameworks still require post-bronchodilator airflow obstruction for formal COPD labeling (62), yet large cohorts have demonstrated that symptomatic smokers with preserved spirometry already experience exacerbations, activity limitation, and impaired quality of life (61). Such observations suggest that clinically meaningful disease can exist outside spirometric definitions, and that distal airway pathology may contribute to symptom burden even when FEV_1_ remains normal (62). The RETHINC trial further underscored this dissociation, showing that dual bronchodilation did not meaningfully reduce symptoms in tobacco-exposed individuals with preserved lung function, indirectly suggesting that symptom mechanisms may reside beyond reversible large airway caliber and potentially within distal airway dysfunction and gas trapping (62).

The link between SAD and disease evolution in COPD is further supported by imaging studies (55). Parametric response mapping has enabled voxel-wise separation of emphysema from functional small airway disease and has provided evidence consistent with fSAD preceding emphysema progression as disease severity increases (55). Longitudinal analyses indicate that regions classified as fSAD can evolve over time (58), reinforcing the interpretation that the distal airway compartment represents an active and progression-relevant substrate rather than a static descriptor (63).

Mechanistically, the clinical consequences of distal airway dysfunction in COPD are most clearly illustrated by dynamic hyperinflation (64). Exercise-induced hyperinflation imposes severe mechanical constraints on tidal volume expansion and contributes to neuromechanical dissociation and exertional dyspnea (65). Premature expiratory airway closure and heterogeneous lung emptying, which are key features of SAD, provide a coherent physiological explanation for gas trapping and hyperinflation, linking distal mechanics more closely to symptoms than resting FEV_1_ (66). This framework positions SAD as a biologically plausible driver of symptom burden, disease progression, and functional limitation, thereby fulfilling many criteria of a candidate treatable trait (67).

### 4.2 Asthma

In asthma, the relevance of SAD is supported less by direct structural data and more by strong physiological and longitudinal outcome evidence (14, 68, 69). Contemporary mechanistic work consistently identifies distal airway inflammation, remodeling, mucus accumulation, and airway closure as central components of asthma pathophysiology, generating increased peripheral resistance and marked ventilation heterogeneity (68, 70). The diagnostic frameworks of GINA, which emphasize variable symptoms and airflow limitation, implicitly acknowledge that disease burden cannot be reduced to a single spirometric snapshot and that persistent functional abnormalities may exist despite normal FEV_1_.

The most comprehensive evidence comes from the ATLANTIS cohort, which integrated spirometry, lung volumes, impulse oscillometry, multiple-breath washout, and CT densitometry in a large longitudinal population (8). Multiple small airway–related measures, including oscillometric indices, ventilation heterogeneity markers, and mid-expiratory flows, were associated with asthma control and exacerbations in univariate analyses (8, 71). Critically, a composite oscillometry-based ordinal score incorporating R5–R20, AX, and X_5_ independently predicted both asthma control and future exacerbations in multivariable models that included established predictors such as GINA treatment step, prior exacerbation history, blood eosinophil levels, and FEV_1_ (8, 72). When this distal physiological signal was included, the contribution of FEV_1_ to exacerbation prediction was no longer significant, suggesting that oscillometry captures outcome-relevant information that is not represented by conventional spirometry (43). The observed dose–risk relationship, with increasing scores associated with progressively higher exacerbation risk, further supports its clinical interpretability (72).

Additional mechanistic studies reinforce ventilation heterogeneity as a central axis of disease instability. Exacerbation-prone asthma has been associated with worse small airway function, including abnormalities in acinar ventilation heterogeneity and oscillometry-derived reactance (73, 74), consistent with a distal closure phenotype rather than purely central airflow limitation. Imaging work linking air trapping and regional remodeling patterns to future exacerbations provides spatial corroboration of this concept (75), suggesting that regional distal obstruction may contribute to disease variability even when spirometry is relatively preserved (76).

In clinical practice, these insights support a more nuanced approach to patient stratification. When symptoms appear disproportionate to spirometric findings, or when control remains poor despite guideline-directed therapy, evaluation of distal airway function may reveal an otherwise hidden disease dimension (77). Oscillometry, in particular, offers a scalable physiological tool in this context (17), while multiple-breath washout and imaging can provide additional mechanistic depth in selected cases (8). Collectively, the evidence base in asthma suggests that SAD has moved from a theoretical concept to a clinically relevant phenotype with demonstrated prognostic value (67).

### 4.3 Fibrotic ILD

In fibrotic ILD, the small airways have long been underappreciated, as traditional disease models primarily focused on alveolar-interstitial injury and fibrotic remodeling, while clinical monitoring largely centered on global measures such as FVC decline, DL_CO_ reduction, symptom progression, and high-resolution CT pattern evolution. However, accumulating structural and physiological evidence is gradually reshaping this view (9). *In vivo* imaging studies using EB-OCT have demonstrated that, even in early IPF with only mild functional impairment, bronchiole density can be significantly reduced (10). Importantly, this loss is observed not only in clearly affected regions but also in areas that appear relatively preserved on conventional imaging (10), suggesting that small airway involvement may occur early in the disease process rather than emerging solely as a secondary consequence of advanced fibrosis (18).

From a mechanical standpoint, fibrosis fundamentally alters the tractional relationship between airways and the surrounding parenchyma, influencing airway caliber and closure behavior (11). These changes may manifest in oscillometric measurements as patterns distinct from classical obstructive disease, forming what has been described as a fibrosis-associated mechanical phenotype (78). Early work has linked reactance-based parameters to prognosis and survival (11, 47), indicating that distal mechanical abnormalities may carry risk-stratification value even when global metrics such as FVC primarily reflect overall lung volume restriction (11, 78).

Despite these emerging signals, important gaps remain compared with the more mature evidence base in COPD and asthma (79). The degree of small airway involvement likely varies across ILD subtypes (24), and the extent to which distal airway metrics change with antifibrotic therapy or track with radiological progression is still unclear (80). Establishing causal links between evolving SAD markers and clinical outcomes will require carefully designed longitudinal studies. This uncertainty, however, also highlights a major research opportunity: if distal airway injury contributes to early disease evolution, then its measurement could provide insight into previously under-recognized aspects of fibrotic disease biology.

From a cross-disease perspective, incorporating SAD into fibrotic ILD frameworks carries important conceptual implications. It challenges the traditional view of fibrosis as a purely parenchymal disorder and offers a potential explanation for variability in symptoms, exercise limitation, and physiological impairment that is not fully captured by conventional lung function metrics. More broadly, it opens the possibility that the distal airway compartment may represent a modifiable component of disease in at least a subset of patients, a hypothesis that remains to be tested in future studies.

## 5 Treatable trait

The concept of SAD as a shared, early, and measurable physiological trait naturally raises a translational question: can this distal compartment be meaningfully targeted, modified, and followed over time in a way that improves clinical outcomes? In the evolving precision-medicine paradigm in respiratory disease, the notion of “treatable traits” has shifted attention away from diagnostic labels alone toward identifiable biological and physiological characteristics that transcend disease boundaries (81). In this context, SAD possesses several key attributes of a candidate treatable trait: it is measurable using multiple techniques, linked to symptom burden and disease instability, and mechanistically connected to key processes such as airway closure, ventilation heterogeneity, and gas trapping. However, the strength of evidence supporting its modifiability varies substantially across COPD, asthma, and fibrotic ILD, reflecting differences in underlying pathology, reversibility, and therapeutic targets.

A useful framework for considering the treatability of SAD can be organized around three interrelated questions. First, can therapies reliably reach the distal airways? Second, can they meaningfully alter inflammatory or structural processes within this compartment? Third, do such modifications translate into measurable physiological or clinical benefit? Addressing these dimensions helps clarify where the evidence is strong, where it is emerging, and where it remains speculative.

### 5.1 Reaching the distal airways: deposition, delivery, and adherence

One of the fundamental challenges in targeting the small airways is anatomical (82). Bronchioles below 2 mm in diameter represent an extensive branching network with large cumulative surface area but low individual airflow velocities (83). Drug deposition within this region depends not only on pharmacologic class but also on particle size, inhaler device characteristics, inspiratory flow profiles, and patient technique (84). Historically, much of inhaled therapy was optimized for central airway deposition, raising the possibility that distal inflammation and closure remained relatively undertreated even when patients were receiving guideline-directed therapy.

The development of extra-fine particle formulations has been a major advance in this regard (83). Particles with a mass median aerodynamic diameter in the range of 1–2 μm are more likely to penetrate into peripheral airways compared with larger particles, particularly under conditions of turbulent flow in proximal bronchi (85, 86). Imaging and deposition studies have demonstrated that such formulations achieve a more homogeneous distribution across central and distal lung regions (87). Although deposition patterns vary between individuals, these data support the biological plausibility that pharmacologic agents delivered in this form may reach and influence the small airway compartment to a greater extent than conventional aerosols (82).

Device selection and inhalation technique further modulate this process (88). Poor inhaler technique, inadequate inspiratory flow, and inconsistent adherence can all disproportionately reduce distal deposition, even when central airway delivery remains adequate (89, 90). In this sense, small airway targeting is not solely a pharmacological issue but also a behavioral and device-dependent one (90). Ensuring optimal inhaler education and adherence may therefore represent an underappreciated component of any strategy aimed at modifying SAD. This is particularly relevant in diseases such as asthma, where distal inflammation and mucus plugging may persist despite apparently appropriate treatment, potentially reflecting incomplete peripheral delivery rather than pharmacologic failure (90).

In clinical practice, persistent symptoms or exacerbations despite guideline-directed therapy should prompt evaluation of inhaler technique and consideration of switching to extra-fine particle formulations to enhance small airway targeting (91).

### 5.2 Treating distal inflammation and remodeling: differential reversibility across diseases

The second dimension concerns whether processes occurring in the distal airways are biologically modifiable. Here, important disease-specific differences emerge.

In asthma, the case for treatability is strongest (67). Distal airway inflammation, mucus accumulation, and airway wall remodeling are central to disease pathophysiology and are, at least in part, responsive to anti-inflammatory therapy (19). Small airway involvement is prevalent across severity spectra and has been linked to poor symptom control, exacerbation susceptibility, and disease instability (44, 92, 93). The observation that distal physiological abnormalities may persist despite normal FEV_1_ has reinforced the idea that targeting the peripheral compartment could improve outcomes not captured by traditional spirometric metrics (72). Inhaled corticosteroids and combination therapies delivered as extra-fine particles have been associated in several studies with improvements in physiological markers related to distal airway function, including oscillometric indices and ventilation heterogeneity measures (94, 95). While the magnitude and consistency of these effects vary, the broader interpretation is that distal inflammation and closure are, to some degree, modifiable (96). Biologic therapies targeting type 2 inflammation may also influence distal airway processes indirectly by reducing eosinophilic inflammation and mucus production, though their specific impact on small airway physiology remains an area of active investigation (97). Collectively, these findings support the positioning of SAD in asthma as a clinically relevant and potentially modifiable phenotype (67).

In COPD, the situation is more complex (46). Distal airway inflammation, bronchiolar narrowing, and structural loss are central to disease development and progression, yet the degree of reversibility is more limited. Bronchodilators can reduce air trapping and dynamic hyperinflation, likely by improving airway caliber and reducing closure tendencies (98). These effects can translate into meaningful improvements in dyspnea and exercise tolerance (99), even when changes in FEV_1_ are modest. In this sense, targeting distal mechanics may influence symptoms through deflation and improved ventilatory mechanics rather than through reversal of structural damage. Anti-inflammatory strategies in COPD have more variable effects. While inhaled corticosteroids can reduce exacerbations in selected patients, their impact on distal structural remodeling is uncertain.

The Chronic bronchitis phenotype, characterized by persistent cough and sputum production, represents a subset of COPD patients with particularly severe small airway involvement (100). Mucus hypersecretion and plugging in the distal airways contribute significantly to ventilation heterogeneity, gas trapping, and exacerbation risk in these patients (101–103). Emerging evidence suggests that mucolytic therapies and agents targeting mucus hypersecretion may preferentially benefit this phenotype by reducing distal airway obstruction and improving SAD-related physiological parameters (104, 105). Thus, in COPD, the treatability of SAD may lie less in reversing structural loss and more in modifying closure behavior, reducing gas trapping, and alleviating mechanical constraints on ventilation.

Fibrotic ILD presents the greatest uncertainty. Structural evidence increasingly suggests that small airway abnormalities and bronchiolar loss may occur early in the disease course (9, 10), but whether these processes are reversible or modifiable remains unclear. Antifibrotic therapies slow decline in global lung function (106, 107), but their effects on distal airway structure and mechanics are largely unexplored. It is plausible that by altering fibrotic progression, these therapies might indirectly influence airway–parenchymal traction relationships and distal mechanics, yet direct evidence is lacking (79). At present, SAD in fibrotic disease can be considered a measurable and potentially prognostically relevant feature, but its status as a modifiable trait remains hypothetical.

### 5.3 Modifying distal mechanics: beyond inflammation toward mechanical phenotypes

The third dimension of a treatable trait framework concerns the possibility of modifying not only inflammatory activity but also the mechanical consequences of distal airway dysfunction. Across diseases, premature airway closure, ventilation heterogeneity, and gas trapping emerge as common physiological pathways linking distal pathology to symptoms and functional limitation.

In COPD, bronchodilation can reduce dynamic hyperinflation, improve ventilatory efficiency, and alleviate exertional dyspnea, even when spirometric changes are small (98). These effects suggest that altering distal mechanics, rather than restoring normal airflow per se, may be a key therapeutic goal. Strategies aimed at reducing closure tendency and improving regional ventilation distribution could therefore represent meaningful endpoints, particularly in patients whose symptom burden is disproportionate to spirometric impairment.

In asthma, therapies that reduce distal inflammation and mucus plugging may stabilize ventilation distribution and decrease closure-related instability, potentially reducing exacerbation risk (14). Physiological measures such as oscillometry and multiple-breath washout may be especially valuable in this context as intermediate endpoints that capture changes in the distal compartment not reflected in FEV_1_ (72).

In fibrotic ILD, the concept of modifying distal mechanics is more speculative but potentially important. Fibrosis alters tissue stiffness and airway tethering, creating a distinct mechanical environment in which airway caliber and closure behavior are governed by complex tractional forces. If distal airway abnormalities contribute to regional ventilation heterogeneity or symptom burden, therapies that influence fibrotic remodeling might indirectly modify these mechanical signatures. Whether such changes can be detected through oscillometric reactance or other mechanics-based indices, and whether they correlate with meaningful clinical outcomes, remains an important area for future research (11, 47, 108).

### 5.4 Integrating SAD into a cross-disease treatable trait paradigm

Current evidence suggests that SAD meets multiple criteria for treatable traits in chronic lung disease, though not all (109). In asthma, there is a close physiological and clinical link, with emerging evidence indicating that distal inflammation and closure can be modulated through targeted therapy (14, 67, 110). In COPD, the distal region is a key driver of symptoms and disease progression; although structural damage may be difficult to reverse, the mechanical consequences such as gas trapping and hyperinflation are clearly modifiable (111). In fibrotic ILD, structural and physiological signals point toward early distal involvement, but intervention-grade evidence remains limited (9, 10, 47).

Importantly, the value of framing SAD as a treatable trait may extend beyond specific therapies. It offers a conceptual bridge between symptoms, physiology, and structure, helping to explain why patients can experience substantial disease burden despite relatively preserved spirometry. It also provides a rationale for integrating additional physiological tools into clinical assessment, particularly in patients whose clinical presentation is not adequately explained by conventional measures (Table 2).

**Table 2:** SAD clinical assessment practical pathway.

Clinical scenario	Preferred initial assessment	Further evaluation if abnormal	Clinical decision implication
Respiratory symptoms with normal conventional spirometry	Oscillometry (IOS/FOT)	If IOS abnormal: add expiratory CT for air trapping; if high asthma suspicion: add MBNW for ventilation heterogeneity	Identify “occult SAD” explaining symptom-spirometry mismatch
Poorly controlled asthma (GINA step 3–4)	Oscillometry + FEV_3_/FEV_6_	If severe SAD indicated: consider switching to extra-fine particle formulations; assess inhaler technique	Guide treatment adjustment to improve distal airway targeting
Symptomatic smokers with preserved FEV_1_/FVC	FEV_3_/FEV_6_ + oscillometry	If abnormal: add expiratory CT + PRM to distinguish functional SAD from early emphysema	Identify pre-COPD patients for early intervention
IPF patients with symptoms disproportionate to FVC decline	Oscillometry (focus on X_5_ and AX)	If abnormal: consider EB-OCT for small airway structural assessment (research setting)	Refine prognostic assessment and identify high-risk patients

GINA, Global Initiative for Asthma; FEV, forced expiratory volume.

Ultimately, the success of SAD as a treatable trait will depend on longitudinal evidence demonstrating that modifying distal airway abnormalities leads to meaningful improvements in outcomes. This will likely require studies that incorporate sensitive physiological endpoints, imaging markers, and patient-centered measures such as dyspnea and activity tolerance. As the field moves toward more individualized management strategies, the distal airway compartment may emerge as a key physiological domain linking early disease processes to clinical expression and therapeutic opportunity.

## 6 Conclusion and future directions

This review has centered on a single overarching question: whether SAD represents a shared, early, measurable, and potentially modifiable physiological feature across COPD, asthma, and fibrotic ILD. The available evidence suggests that the distal airway compartment exhibits strikingly consistent patterns of functional abnormality across these conditions and plays a meaningful role in symptom generation, disease heterogeneity, and progression. At the same time, the extent to which these abnormalities are reversible, and therefore clinically modifiable, varies substantially between diseases.

In asthma, the evidence is strongest. Distal airway inflammation, mucus plugging, and a tendency toward airway closure are closely linked to poor symptom control and increased exacerbation risk, and they appear at least partially responsive to anti-inflammatory treatment. As a result, SAD has increasingly emerged as a clinically relevant functional phenotype that helps explain disease instability beyond what is captured by spirometry alone. In COPD, by contrast, small airway injury is more structural and persistent in nature. The therapeutic value of targeting this compartment lies less in reversing damage and more in modifying its mechanical consequences, particularly gas trapping and dynamic hyperinflation, which are key contributors to dyspnea and exercise limitation. In fibrotic ILD, structural and physiological evidence of distal airway involvement is steadily accumulating, but whether these abnormalities can be directly modified remains uncertain. At present, SAD in fibrotic disease may be best regarded as a physiologically meaningful and potentially prognostic signal rather than a clearly actionable therapeutic target.

From a cross-disease perspective, SAD is perhaps best conceptualized not as a disease-specific marker, but as a functional phenotype reflecting abnormalities within the “terminal ventilatory unit.” This framework offers a coherent explanation for the long-recognized mismatch between symptom burden and spirometric findings and supports the idea that SAD may represent an early and clinically important component of chronic lung disease.

Several key knowledge gaps remain. First, there is still no universally accepted physiological definition or standardized threshold for SAD, which limits comparability across studies and hinders clinical implementation. Standardization will be essential if distal airway measurements are to be used for risk stratification, longitudinal monitoring, or as endpoints in clinical trials. Second, although associations between distal airway abnormalities and symptoms, exacerbations, and functional impairment are increasingly well established, longitudinal interventional evidence demonstrating that modifying SAD leads to sustained clinical benefit remains limited. Third, the relationship between structural injury and functional impairment is not uniform across diseases, and the mechanisms linking distal pathology to measurable physiological signals require further clarification.

Future research should therefore focus on developing standardized assessment frameworks, establishing large longitudinal cohorts integrating imaging and physiological data, and incorporating distal airway metrics as intermediate endpoints in clinical trials. Such approaches will be essential to determine whether targeting this compartment can translate into meaningful improvements in dyspnea, activity tolerance, and disease progression.

From a clinical perspective, the recognition of SAD as a shared early trait has immediate practical implications. Oscillometry, in particular, offers a simple, effort-light, and scalable method for assessing distal airway function in routine clinical practice, requiring only quiet tidal breathing and minimal patient cooperation. Unlike more complex techniques such as MBNW or EB-OCT, oscillometry is increasingly available in primary and secondary care settings, making it feasible to integrate SAD assessment into standard respiratory disease management. By identifying patients with isolated distal airway dysfunction who would otherwise be missed by conventional spirometry, clinicians can implement earlier interventions and more personalized treatment strategies, potentially improving symptom control and reducing disease progression.

Overall, SAD is shifting from a historically overlooked peripheral abnormality to a central lens through which the heterogeneity of chronic lung diseases can be better understood. In asthma, it already represents a clinically relevant and potentially modifiable phenotype; in COPD, it underpins key mechanical drivers of symptoms and progression; and in fibrotic lung disease, it is emerging as a newly recognized pathophysiological aspect. As measurement techniques continue to advance and therapeutic strategies evolve, the distal airway compartment is likely to become an increasingly important focus in both research and clinical management. In this context, SAD may ultimately be established as a core cross-disease functional phenotype linking early pathological processes to clinical expression and therapeutic opportunity.
